# Multifunctional action of *Bacillus subtilis* for *Fusarium verticillioides* biocontrol and growth promotion in corn seeds

**DOI:** 10.1007/s42770-026-02080-x

**Published:** 2026-09-11

**Authors:** Melissa Tiemi Hirozawa, Mario Augusto Ono, Igor Massahiro de Souza Suguiura, Amanda Kaori Sugahara, Maria Inês Rezende, André Luiz Martinez de Oliveira, Elisabete Yurie Sataque Ono

**Affiliations:** 1 Department of Biochemistry and Biotechnology, State University of Londrina, P.O. box 10.011, Londrina, Paraná 86057-970 Brazil; 2 Department of Immunology, Parasitology and General Pathology, State University of Londrina, P.O. box 10.011, Londrina, Paraná 86057-970 Brazil

**Keywords:** Antagonism, Antifungal compounds, Toxigenic fungi, Biofungicide, Auxins

## Abstract

This study aimed to evaluate the in vitro antifungal activity and corn seed protective effect of two *Bacillus subtilis* isolates in two formulations, against *Fusarium verticillioides*, along with their indole acetic acid, siderophore production, and phosphate solubilization ability. Antifungal activity of two *Bacillus subtilis* isolates (NT1 and NT2) was investigated against *F. verticillioides* 97L in solid media and on corn seeds. Both *B. subtilis* isolates showed antifungal activity through production of diffusible antifungal compounds and NT2 showed the highest radial growth inhibition (61.7%). The seed treatments with *B*. *subtilis* based formulations, powder (P) and suspension (S), reduced the disease severity index compared to the control (*P* < 0.05), therefore they were efficient in inhibiting *F. verticillioides* 97L growth in corn seed and had no negative effects on seed germination and vigor. Concerning the vigor test, treatments with *B. subtilis* NT1(P) and NT2 (P) demonstrated a significant (*P* < 0.05) increase in shoot length and biomass production in comparison to the untreated seeds, which can be related to the ability of these isolates to produce indole acetic acid and to solubilize phosphate. None of the isolates produced siderophores. The *B. subtilis* isolates (NT1 and NT2) are promising biocontrol agents for developing new multifunctional formulations intended for management of corn disease caused by *F. verticillioides* as an alternative to chemical fungicides. Despite the efficacy observed in the current study, further research is required to evaluate the shelf-life and stability of the powder and suspension formulations under various storage conditions.

## Introduction

Corn (*Zea mays L*.) is the most produced cereal in the world. Production in the 2024/25 harvest season was 141.1 million metric tons in Brazil and 1.2 billion metric tons worldwide [1, 2]. Despite the good results in grain production, the diseases caused by fungi, especially *Fusarium verticillioides*, are one of the main limiting factors of this crop. In addition to decreasing yield, they often result in low-quality grain due to contamination with mycotoxins [3].

*Fusarium verticillioides* is the main corn phytopathogen, associated with diseases that affect the various stages of the plant, including diseases in the seed, stem, root, ear rot, deterioration during storage, in addition to grain contamination by fumonisins [4]. Fumonisins are toxic secondary metabolites and comprise a group of 28 analogues, and fumonisin B_1_ (FB_1_) is the most toxic and prevalent in corn. Consumption of fumonisin contaminated corn and corn-based products has been associated with increased risk of esophageal cancer and neural tube defects in humans, leukoencencephalomalacia (LEM) in horses and pulmonary edema in swine [5–8]. The International Agency for Research on Cancer (IARC) classified FB_1_ as a group 2B, that is possibly carcinogenic to humans [9].

Chemical fungicides are the main control strategy for *F. verticillioides* in crops [10]. However, in addition to the possible health problems caused by exposure to these compounds, the overuse of fungicides can cause environmental problems, such as disruption of ecological balance, which is linked to decreased soil health [11]. In this context, several studies have been conducted aiming to develop sustainable solutions for plant pathogen management [12, 13]. Biological control using antagonist microorganisms is considered one of the most promising alternatives [14]. Antagonistic bacteria can suppress the phytopathogenic fungi through antibiosis, production of lytic enzymes and other bioactive metabolites (cyclic lipopeptides, volatile organic compounds), competition for nutrients and ecological niches, and induction of plant defense responses [15]. To date, studies on the biological control of phytopathogenic fungi have demonstrated the antagonistic capacity of various microorganisms against important phytopathogens in agriculture [13, 16]. Among them, representatives of the *Bacillus* genus provide the potential for biocontrol agents due to their ability to produce a wide range of antifungal compounds such as hydrolytic enzymes (cellulases, proteases, and β-glucanases), antimicrobial peptides (lipopeptides), siderophores and volatile organic compounds [15, 17]. Some authors have reported that species such as *Bacillus amyloliquefaciens* FZB42 have more than 8.5% of the genome involved with the synthesis of compounds associated with biocontrol action [18]. In addition to the direct bioprotective effect, *Bacillus* species act through indirect mechanisms, producing compounds that stimulate plant growth (indoleacetic acid, siderophores, phosphate solubilizers) and inducing plant systemic resistance [19]. Moreover, *Bacillus* genus can grow rapidly and sporulate, which are important characteristics for commercial formulation development [20]. Therefore, the objectives of this study were to evaluate the antifungal activity of two *B. subtilis* isolates against *F. verticillioides* and the effect of *B. subtilis*-based formulations on the physiological (seed germination percentage and vigor) and disease severity index of corn seeds, as well as their ability to produce indole acetic acid, siderophores and solubilize phosphate.

## Material and methods

### Microorganisms and growth conditions

*Fusarium verticillioides* 97L was provided by the Mycological Culture Collection of the Food Science and Technology Department, State University of Londrina, Paraná, Brazil. It was grown on potato dextrose agar (PDA) for 7 days at 25 ºC. Conidia suspensions were prepared in 0.85% sodium chloride solution with 0.1% Tween 80.

The *Bacillus subtilis* isolates, NT1 and NT2, were obtained from the Microbiological Culture Collection of the Department of Immunology, Parasitology and General Pathology, State University of Londrina, Paraná, Brazil. These bacteria were originally isolated from Nattō, a traditional Japanese food made from fermented whole soybeans that is available in grocery stores in Londrina, Northern Paraná State, Brazil [21]. For storage, *B. subtilis* isolates were cultured in Nutrient Agar (NA) tubes, incubated at 37 °C for 24 h and stored at 5 °C. For antagonism assays, *B. subtilis* isolates were cultured in Luria–Bertani (LB) broth at 37 °C and 150 rpm for 24 h.

### Evaluation of bacterial antagonistic activity

A dual culture assay was performed to assess bacterial antagonistic activity [22]. Agar plugs (5 mm) of *Fusarium verticillioides* 97L mycelium from a 7-day-old culture were placed on PDA (20 mL), 2 cm away from the periphery of a Petri dish (100 mm × 20 mm). Next, 10 µL of *Bacillus subtilis* NT1 or NT2 (10^8^ CFU mL^−1^) were inoculated and spread 2 cm away from the opposite edge of the Petri dish. As a control, an agar plug (5 mm) of *F. verticillioides* 97L mycelium was placed in the same manner on a fresh PDA plate. After incubation at 25 ºC for 7 days, the mycelial growth radius was measured, and the inhibition percentage (I%) was calculated using the formula:

$$\mathrm{I}\mathrm{\%}=\left[\left({\mathrm{D}}_1-{\mathrm{D}}_2\right)/{\mathrm{D}}_1\right]\;\mathrm x\;100$$

where D_1_ is the mycelial growth radius in the control (without *B. subtilis*), and D_2_ is the mycelial growth radius in the treatment. The appearance of an inhibition zone indicates the production of diffusible compounds.

All experiments were performed with three independent biological replicates.

### Preparation and evaluation of bioformulation

#### Formulations based on *B. subtilis* endospores

To prepare the endospore suspension, 10% (v/v) of *B. subtilis* NT1 or NT2 (10^6^ CFU mL^−1^) inoculum were inoculated in Difco sporulation medium (DSM) at 37 ºC, 180 rpm for 72 h [23]. Endospores were collected by centrifugation, washed in 0.9% (v/v) saline solution and resuspended in glucose solution (1 g L^−1^). Then, the endospore suspension was incubated in a water bath at 80 °C for 10 min to eliminate the vegetative cells. Viable endospores were quantified by serial dilution, plating in LB agar followed by incubation at 37 ºC for 24 h. The *B. subtilis* powder formulation was prepared by mixing the endospore suspension and a mixture of inorganic carriers (25 g talc, 5 g carboxymethylcellulose, 7.5 g CaCO_3_ and 1.25 g glucose). The *B. subtilis* endospore suspension formulation was achieved by diluting the endospores in a vehicle (2 g L^−1^ carboxymethylcellulose, 2 g L^−1^ yeast extract, 1 g L ^−1^ glucose, 1 g L ^−1^ xanthan gum, 1 g L ^−1^ KCl, 0.12 g L ^−1^ MgSO_4_. 7H_2_O, 8 mL Tween 20 and 1 L H_2_O qsp). The final concentration of the powder and suspension formulations was 10^8^ CFU g^−1^ and 10^8^ CFU mL^−1^, respectively. Before mixing with the endospore suspension, all the components of each formulation were homogenized and autoclaved at 121 ºC for 15 min. Controls were prepared using formulations without *B. subtilis* endospores [24].

#### Seed treatment

The experiments were performed using untreated organic corn seeds (*Zea mays* L.), BRS Caimbé, EMBRAPA. The seeds were sanitized with 1% hypochlorite solution under stirring for 3 min followed by washing with autoclaved distilled water [25]. Then, the seeds were placed on sterile Petri dishes containing filter paper to remove the excess residual water. For the treatment, the corn seeds (85 g) were placed in sterile plastic bags and mixed with the *B. subtilis* formulations (2 ml suspension or 2 g powder) until the seeds were covered.

#### Germination percentage and vigor test

The germination percentage and vigor test were performed to evaluate the influence of the *B. subtilis* treatment on the corn seed physiological quality. The germination percentage was performed with four replicates of 50 seeds for treatment and control. After the *B. subtilis* treatment, the seeds were distributed on 2 sheets of germitest paper moistened with distilled water at the proportion of 2.5 times the mass (g) of the paper, forming 5 rows containing 10 seeds. Then, they were covered with 1 sheet of moistened germitest paper and rolled up (in the form of paper rolls), packed in plastic bags, held in a chamber-type germinator, in an upright position, at 25 °C, for 7 days [26]. Evaluations were performed after seven days, in which the number of seeds that produced seedlings classified as normal was determined [25]. The vigor test was performed on four replicates of 20 seeds for each treatment. Evaluations were performed on the seventh day, when the primary root and shoot length and the dry weight were determined only for the normal seedlings. The dry weight was determined by placing the shoot and root in paper bags in an air circulation drying oven (60 °C) until constant mass. The total mass was divided by the number of normal seedlings, obtaining the average dry seedling biomass (mg seedling^−1^).

#### Efficacy of the bacterial treatments against an artificial infection with *F. verticillioides*

The efficacy of the bacterial treatments against an artificial *F. verticillioides* infection was evaluated using 4 replicates of 50 corn seeds per treatment. First the seeds were sanitized as previously described and were then incubated at 25 ºC for 24 h and then at −20 ºC for 24 h to reduce the germination process. After incubation, the seeds were treated with *B. subtilis* formulations as previously described and inoculated with 30 μL *F. verticillioides* suspension (10^5^ conidia mL^−1^). The seeds were distributed 1 to 2 cm apart on a germitest paper layer (3 units) moistened with distilled water at 2.5 times the mass (g) of the dried paper. The plates containing the seeds were kept in chamber type germinator at 25 ºC with a 12 h photoperiod for 5 days. The seeds were evaluated individually by checking the appearance of characteristic fungal growth (seed rot) and were scored on a 0–5 point scale according to Machado et al.: 1—seeds without visible fungal growth; 2—mycelium covering up to 25% of the seed; 3—mycelium covering from 25 to 50% of the seed; 4—mycelium covering from 50 to 75% of the seed; 5—mycelium present on more than 75% of the seed surface [27]. The results were converted to a disease severity index adapted from root rot analysis according to the formula [28]:

$$\mathrm{Disease}\;\mathrm{severity}\;\mathrm{index}\;\left(\%\right)=\left[\sum\left(\mathrm R\;\mathrm x\;\mathrm N\right)\;\mathrm x\;100\;\right]/\mathrm H\;\mathrm x\;\mathrm T$$

where R = categories of seed rot, N = number of seeds with these categories, H = highest category, and T = total of seeds.

### Detection of plant growth-promoting traits

#### Indole acetic acid (IAA) production

IAA production was quantified according to Sarwar and Kremer with modifications [29]. The *Bacillus subtilis* isolates were cultured in DYGS broth at 37 ºC, 180 rpm for 48 h. Then, each culture was adjusted to 10^8^ cells mL^−1^ concentration and a 100 μL aliquot of the culture was inoculated in DYGS broth supplemented or not with 0.1% L-tryptophan at 37 ºC, 150 rpm for 48 h. Then, the cultures were centrifuged at 8,000 × *g* for 5 min. One mL of the supernatant was mixed with 2 mL of modified Salkowsky reagent (40 mmol L ^−1^ FeCl_3_; 7.9 mol L ^−1^ H_2_SO_4_) and incubated in the dark for 30 min. Absorbance was measured at 530 nm in a UV–VIS spectrophotometer Biochrom Libra S22. The IAA concentration was calculated from a standard curve obtained in the range from 5 to 30 μg mL^−1^ IAA. All the experiments were performed in triplicate.

#### Phosphate solubilization

The ability of the isolates to solubilize inorganic iron phosphate (FePO_4_. 2H_2_O) was evaluated using the liquid culture medium NBRIP, supplemented with iron phosphate (FePO_4._ 2H_2_O) at a final phosphorus (P) concentration of 0.1% (m/v) [30]. The initial pH of the medium was adjusted to 7.0 before autoclaving at 121 °C for 20 min. Phosphate solubilization was quantified in 50 mL Falcon tubes containing 10 mL NBRIP medium inoculated in triplicate with 100 μL cultures previously grown at 37 °C for 48 h under constant shaking at 180 rpm in 5 mL DYGS broth, adjusted before the start of fermentation to an OD600 of 0.3 or a concentration of 10⁸ cells mL^−1^. The tubes were incubated at 37 °C for 7 days at 180 rpm. Cultures were centrifuged at 8,000 x*g* for 5 min, and the supernatant was used for quantitative phosphorus analysis.

Soluble phosphorus was assessed using the modified phosphomolybdic method described by Murphy and Riley [31]. In a test tube, 1 mL of the supernatant was mixed with 2 mL of a diluted ammonium molybdate solution and 20 μL of a 5% ascorbic acid solution. After 30 min, absorbance at 660 nm was measured in a spectrophotometer. Measurements were performed in triplicate. The calibration curve was constructed in the range from 0.5 to 4.0 mg P L^−1^) of a standard KH_2_PO_4_ solution (25 mg P L^−1^).

#### Siderophore production

Siderophore production was evaluated using a modified chrome azurol S (CAS) assay according to Schwyn and Neilands [32]. *Bacillus subtilis* NT_1_ and NT_2_ were cultured in 5 mL of DYGS broth at 37 °C under constant shaking at 180 rpm for 48 h. To ensure strict iron-deficient conditions for siderophore production, an iron-deficient T-CAS agar medium was used, and all glassware was pre-rinsed with 6.0 mol L^−1^ HCl to remove trace iron contamination. Then, 5 μL (10^8^ cells mL^−1^) of each isolate was spotted onto the center of chrome azurol sulphonate (T-CAS) agar plates and incubated at 37 °C for 5 days. As positive control, a known siderophore-producing *Streptomyces* sp. BTM287 strain [33] was used. The formation of a yellow or orange halo around the colonies indicated the ability to produce siderophores.

### Statistical analysis

All data was analyzed statistically by the Statistica software version 7 (Stat Soft, Inc., Tulsa, OK, USA, 2007). The effect of *Bacillus subtilis* NT1 and NT2 isolates on the *F. verticillioides* 97L mycelial growth was analyzed by the Student t test. Corn seed physiological and health quality data were analyzed using ANOVA with the Duncan multiple range test (*P* < 0.05). Differences in the mean IAA production and phosphate solubilization between the two *B*. *subtilis* isolates were analyzed using ANOVA followed by the Tukey test (*P* < 0.05).

## Results

### Bacterial antagonistic activity

The dual culture assay was performed to assess the inhibitory effect of living bacterial cultures (*B. subtilis* NT1 and NT2) on fungal growth. The two *B. subtilis* isolates were able to inhibit *F*. *verticillioides* 97L mycelial growth, and *B. subtilis* NT2 showed the highest percentage of inhibition (61.7 ± 1.5%) compared to *B. subtilis* NT1 (52.3 ± 0.6%; *P* < 0.05).

### Efficacy of bacterial treatments against *Fusarium verticillioides* infection and their impact on corn seed physiological quality

**Fig. 1:**
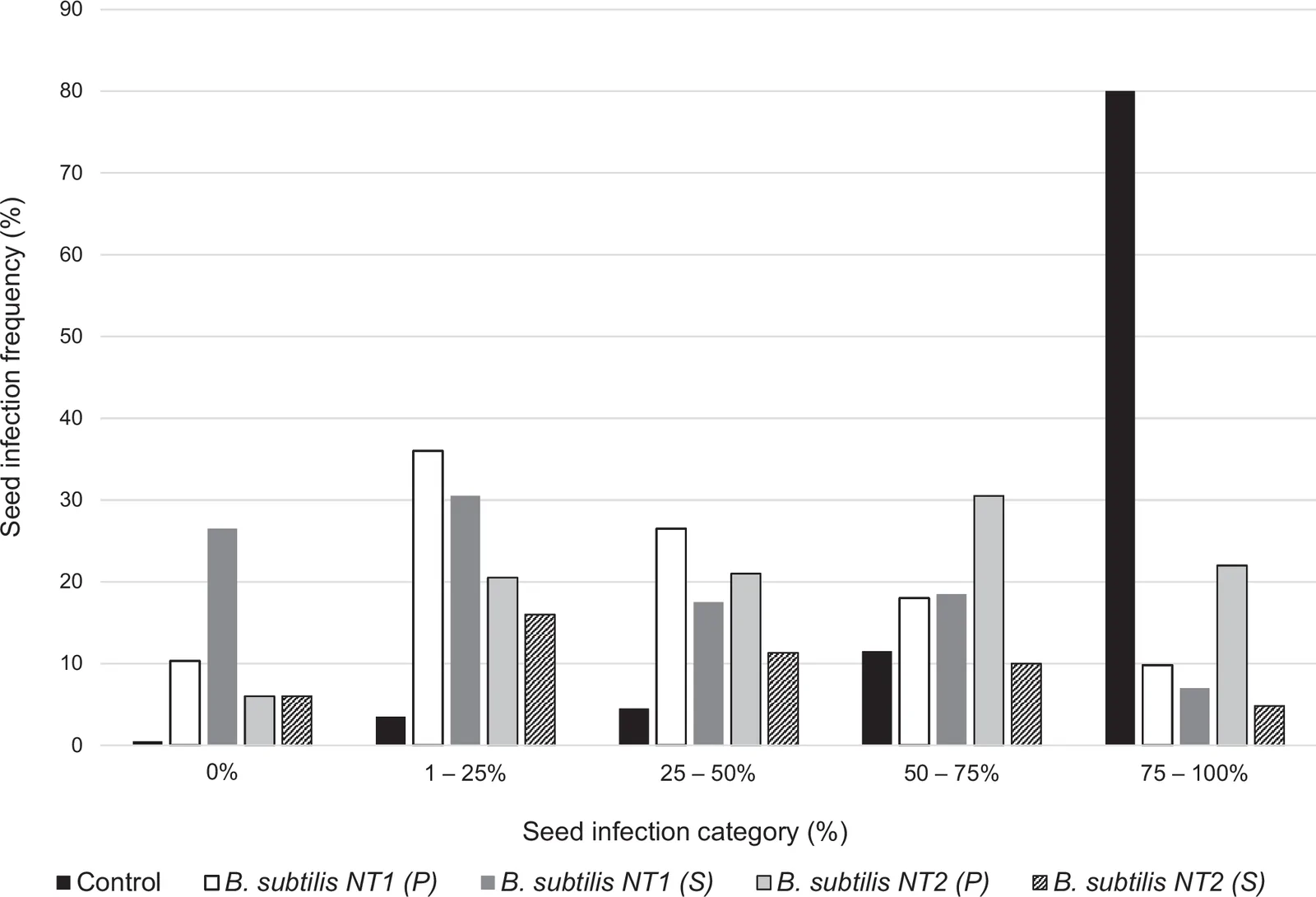
Seed infection frequency (%) and seed infection category (%) by *F. verticillioides* 97L in corn seeds treated with *Bacillus subtilis* powder (P) and (suspension) based formulations

All *B. subtilis* formulations significantly reduced the disease severity index compared to the control (*P* < 0.05, Table 1) showing a protective effect of the bacterium inoculation against *F. verticillioides* 97L. Moreover, a decrease in the incidence of grains with higher infection severity was observed (Fig. 1). In untreated corn seeds (control), the highest infection severity (> 75%) was detected in 80% of the seeds, while seeds treated with *B. subtilis* NT1 (S), *B. subtilis* NT1(P), and *B. subtilis* NT2 (S) showed 7%, 9%, and 4.8% of the seeds with the highest infection severity (Fig. 1).

**Table 1:** Effect of seed treatment with *Bacillus subtilis* based formulations on corn seed physiological quality (germination percentage and vigor) and against an artificial infection with *F. verticillioides* (disease severity index) (cultivar BRS Caimbé)

	Physiological quality				Disease severity index (%)
Treatments	Seed germination (%)	Root length (cm)	Shoot length (cm)	Biomass production (mg seedling^−1^)	
Control	98.0 ± 0.1^a^	11.0 ± 0.5^a^	10.7 ± 0.4^b^	0.051 ± 0.001^c^	91.75 ± 7.0^a^
*Bacillus* sp. NT1 (P)	96.5 ± 2.6^a^	9.0 ± 0.8^b^	12.8 ± 1.2^a^	0.060 ± 0.002^a^	44.00 ± 5.0^c^
*Bacillus* sp. NT1 (S)	94.0 ± 3.4^a^	9.5 ± 0.6^b^	10.8 ± 0.8^b^	0.053 ± 0.002^bc^	36.62 ± 5.4^c^
*Bacillus* sp. NT2 (P)	96.0 ± 2.5^a^	9.2 ± 0.9^b^	13.0 ± 0.9^a^	0.062 ± 0.001^a^	60.37 ± 4.2^b^
*Bacillus* sp. NT2 (S)	95.0 ± 3.0^a^	9.5 ± 0.4^b^	11.0 ± 0.4^b^	0.049 ± 0.004^c^	43.75 ± 6.0^c^

*P* powder formulation; *S* suspension formulation; *Control* untreated seeds; Means followed by different lower-case letters in the same column are significantly different by the Duncan multiple range test (*P* < 0.05)

Seed treatment with *B. subtilis* NT1 or NT2 did not affect germination (Table 1), which did not differ significantly from the control (*P* > 0.05). Concerning the vigor test, treatments with *B. subtilis* NT1 (P) and *B. subtilis* NT2 (P) demonstrated a significant (*P* < 0.05) increase in shoot length and biomass production in comparison to the untreated seeds (Table 1). On the other hand, all *Bacillus subtilis* treatments resulted in a statistically significant decrease in root length compared to the control (*P* < 0.05).

### Detection of plant growth-promoting traits

*B. subtilis* NT1 and *B. subtilis* NT2 produced 0.59 ± 0.14 and 0.42 ± 0.06 μg mL^−1^ IAA (without tryptophan supplementation), respectively, whereas 1.70 ± 0.05 and 2.61 ± 0.10 μg mL^−1^ IAA (with tryptophan supplementation) (Table 2).

**Table 2:** Indole acetic acid (IAA), Phosphate solubilization and siderophore production by *Bacillus subtilis* NT1 and NT2 isolates

Isolates	IAA (μg mL^−1^)		Phosphate solubilization (μg mL^−1^)	Siderophore production
	Without L-tryptophan	With L- tryptophan		
*B. subitilis* NT1	0.59 ± 0.14^aB^	1.70 ± 0.05^bA^	1.32 ± 0.11^a^	-
*B. subitilis* NT2	0.42 ± 0.06^aB^	2.61 ± 0.10^aA^	1.97 ± 0.15^a^	-

Means followed by the same lowercase letter in the same column are not significantly different by the Tukey test (*P* < 0.05)

Means followed by the same uppercase letter in the same line are not significantly different by the Tukey test (*P* < 0.05)

+ : positive

-: negative

The bacterial isolates showed phosphate solubilization ranging from 1.32 ± 0.11 (NT1) to 1.97 ± 0.15 µg mL^−1^ (NT2) (Table 2).

Although siderophore synthesis was evaluated under strict iron-limiting conditions, *B. subtilis* NT1 and *B. subtilis* NT2 did not produce a detectable yellow or orange halo around the colonies (Table 2).

## Discussion

*F. verticillioides* control in corn is essential to reduce productivity losses and serious health risks associated with fumonisin contamination. Biological control using antagonist bacteria has been reported as an efficient and environmentally friendly alternative in the control of phytopathogenic fungi [15]. In this context, bacteria of the genus *Bacillus* stand out for their ability to produce several bioactive compounds (surfactin, iturin, fengycin, cell wall degrading enzymes, volatile organic compounds) capable of inhibiting a broad range of phytopathogens [15]. Some studies have shown the efficacy of several *Bacillus* species in controlling *Fusarium* species growth in in vitro and *in planta* trials, but *Bacillus*-based commercial products against *F. verticillioides* for corn seed treatment are still scarce [16, 34, 35]. In the present study, in vitro assays revealed that both *B. subtilis* isolates were able to inhibit *F. verticillioides* 97L mycelial growth and the clear zone of inhibition indicated the production of diffusible antifungal compounds. A previous study [21] demonstrated that cell-free supernatants (CFSs) from both *B. subtilis* isolates (NT1 and NT2) contained extracellular proteases, biosurfactants (lipopeptides), and polar low molecular weight compounds. These diffusible antifungal compounds likely include cyclic lipopeptides (iturin, fengycin) that contribute to the observed antifungal activity. Cyclic lipopeptides act on the fungal membrane and form ion-permeable pores, which results in ion leakage, osmotic imbalance, and ultimately fungal death [36]. *B. subtilis* isolates NT1 and NT2 were selected primarily because of their high antifungal efficacy (up to 61.7% inhibition)*.*

*Fusarium verticillioides* is a seed-borne phytopathogen that infects corn and can cause several corn diseases at different stages of its development. One of the corn infection routes begins with conidia or hyphae present inside the seeds or on the surface that develop within the plant resulting in systemic corn infection [4]. In this context, seed treatment is an important tool for controlling seed-borne fungi and the microbial seed treatments have been reported as an efficient and environmentally friendly method [37].

Biopesticides based on *Bacillus thuringiensis* and *Bacillus sphaericus* are the most used microbial biopesticides worldwide [38]. Commercially available biopesticides are usually presented as dust, liquid or granules. For seed microbial treatment is commonly applied as powder or liquid [20]. In this study, seed treatments with *B*. *subtilis* based formulations (powder and suspension) reduced the disease severity index compared to the control (*P* < 0.05), therefore they were efficient in controlling *F. verticillioides* 97L growth in corn seed. These data are in line with Martínez-Álvarez et al. [39], who used powder formulation based on *Bacillus cereus *sensu lato strain B25 in corn seed treatment. The results showed suppression of *F. verticillioides* P03 growth with a significant reduction in the root rot disease index, and no negative effect on seed germination. In another study by Lizárraga-Sánchez et al. [34], corn seed inoculation with *Bacillus cereus *sensu lato strain B25 controlled the *Fusarium* stalk rot (FSR) and *Fusarium* ear rot (FER) incidence and disease severity in corn [34].

Although some biocontrol agents show high in vitro and in vivo inhibition, they can negatively affect seedling development. Therefore, seed growth parameters, including germination percentage and vigor tests, were investigated after *B. subtilis* NT1 and NT2 application. Considering that seed treatment with *B. subtilis* NT1 or NT2 did not affect germination and significantly increased shoot length and biomass production compared to untreated seeds (*P* > 0.05), plant growth-promoting traits were further evaluated.

In addition to growth inhibition of various phytopathogenic fungi, some bacteria of the genus *Bacillus* can induce physiological changes in plant growth. Considering the essential role of phosphorus, indole acetic acid and siderophores in plant development, application of microorganisms that solubilize phosphate, produce IAA and siderophores represent a sustainable alternative to improve the efficiency of plant nutrition. This approach can minimize dependence on chemical fertilizers and mitigate their negative environmental impacts, providing more sustainable and healthy crop development [40].

The IAA production by the NT2 isolate was higher than the NT1 isolate (*P* < 0.05) with tryptophan supplementation, whereas there was no significant difference between the two isolates without tryptophan supplementation by the Tukey test (*P* > 0.05). The IAA production by both *B. subtillis* isolates was low compared to many strains reported in the literature, particularly when tryptophan was supplemented. Balbinot et al. [41] comparing the effect of different *Bacillus* sp. on corn growth reported values of 2 to 34.28 µg mL^−1^ with a mean of 11.90 µg mL^−1^ for 27 analyzed isolates, with 45% of these isolates producing 10 to 19 µg mL^−1^ [41]. Despite these results, the IAA concentration produced by NT1 and NT2 is still able to promote corn growth, in fact both concentrations found in this study fall near the optimal concentration of 0.01 mmol L^−1^, which are related to significant increases in the height, shoot and root biomass of plants [42]. In the same manner, such amount of IAA is also related to modifications in root morphology with increases in root length, tip number and tip density, and enhancing mineral uptake such as calcium and magnesium by corn [42]. Accordingly, these responses to IAA can be related to the increase in shoot length and biomass production observed in this study after seed treatment. On the other hand, all *B. subtilis subtilis* treatments resulted in a statistically significant decrease in root length compared to the control (*P* < 0.05). These results are in line with Calzavara et al. [43], who demonstrated that corn plants inoculated with *Bacillus* sp. strains had shorter roots than uninoculated control plants, while root branching increased. Consequently, there was a significant increase in root biomass, a phenomenon attributed to the hormonal imbalance introduced by bacterial inoculation and establishment in host plant tissues [43, 44].

Considering that nutrient availability such as phosphorus and iron is essential for plant growth, the ability of *Bacillus subtilis* NT1 and NT2 isolates to solubilize phosphate and produce siderophores was evaluated.

Phosphorus is a limiting element for plant growth, because of its predominance in insoluble inorganic form, associated with aluminum, calcium, or iron ions, or in its organic form, which are not absorbed by plants [45]. Bacteria capable of converting insoluble phosphates into soluble ones play a crucial role in supplying P to plants in an environmentally friendly and sustainable way [46]. Both *Bacillus subtilis* NT1 and NT2 isolates solubilized inorganic iron phosphate, and there was no significant difference between the two isolates (*P* > 0.05). The concentrations released were in accordance with other isolates such as *B. subtilis* 1920 (1.34 μg mL^−1^) and *B. subtilis* 1982 (1.73 μg mL^−1^) but lower than *B*. *subtilis* B2084 (57.33 μg mL^−1^) [40].

In addition to solubilizing iron present in the soil, production of siderophores can favor microorganisms to compete effectively against other ones for the iron available in the environment. Under these conditions, the microorganism develops satisfactorily while limiting the availability of iron to its competitors, inhibiting their growth [47]. In this study, neither *B. subtilis* NT1 nor *B. subtilis* NT2 produced siderophores in chrome azurol sulphonate (CAS) agar plates, indicating that these isolates naturally lack the ability to produce siderophores under the tested conditions, rather than experiencing iron suppression. Several factors affect siderophore production such as strain, culture medium composition or cultural conditions (temperature, pH, incubation time) [48].

In summary, *B. subtilis* NT1 and NT2 isolates are promising candidates for use in biocontrol programs for corn protection against diseases caused by *F. verticillioides* in both formulations, powder, and suspension. In addition to suppressing *F. verticillioides* growth, they showed more than one plant growth-promoting feature, IAA and phosphate solubilization, which probably can interact with each other to synergistically improve plant growth, as observed in the enhanced shoot length and biomass production of treated corn. The ability of *B. subtilis* isolates to control *F. verticillioides *in vitro and in situ, associated with their benefits to seedling development, reinforce their potential as biocontrol agents and an alternative to synthetic fungicides. Despite the efficacy observed in the current study, further research is required to evaluate the shelf life of the powder and suspension formulations, including the effects of temperature, packaging, and storage conditions on their stability.

## Data Availability

The data that support the findings of this study are available upon request.
